# Public Health Program for Decreasing Risk for Ebola Virus Disease Resurgence from Survivors of the 2013–2016 Outbreak, Guinea

**DOI:** 10.3201/eid2602.191235

**Published:** 2020-02

**Authors:** Mory Keita, Sakoba Keita, Boubacar Diallo, Momo Camara, Samuel Mesfin, Koumpingnin Yacouba Nebie, N’Faly Magassouba, Seydou Coulibaly, Boubacar Barry, Mamadou Oury Baldé, Raymond Pallawo, Sadou Sow, Amadou Bailo Diallo, Pierre Formenty, Mamoudou Harouna Djingarey, Ibrahima Socé Fall, Lorenzo Subissi

**Affiliations:** World Health Organization Country Office, Conakry, Guinea (M. Keita, B. Diallo, S. Mesfin, K.Y. Nebie, S. Coulibaly, B. Barry, M.O. Baldé, R. Pallawo);; National Agency for Health Security, Ministry of Health, Conakry (S. Keita, M. Camara); University Gamal Abdel Nasser, Conakry (N. Magassouba, S. Sow);; World Health Organization Regional Office for Africa, Brazaville, Republic of the Congo (A.B. Diallo, M.H. Djingarey, I.S. Fall);; World Health Organization, Geneva, Switzerland (P. Formenty);; Global Outbreak Alert and Response Network, Geneva (L. Subissi)

**Keywords:** Ebola, surveillance, Guinea, survivors, West Africa, viruses, resurgence, Ebola virus disease

## Abstract

At the end of the 2013–2016 Ebola virus disease outbreak in Guinea, we implemented an alert system for early detection of Ebola resurgence among survivors. Survivors were asked to report health alerts in their household and provide body fluid specimens for laboratory testing. During April–September 2016, a total of 1,075 (88%) of 1,215 survivors participated in the system; follow up occurred at a median of 16 months after discharge (interquartile range 14–18 months). Of these, 784 acted as focal points and reported 1,136 alerts (including 4 deaths among survivors). A total of 372 (91%) of 408 eligible survivors had >1 semen specimen tested; of 817 semen specimens, 5 samples from 4 survivors were positive up to 512 days after discharge. No lochia (0/7) or breast milk (0/69) specimens tested positive. Our findings underscore the importance of long-term monitoring of survivors’ semen samples in an Ebola-affected country.

The 2013–2016 Ebola virus disease (EVD) outbreak was the largest outbreak since the discovery of Ebola virus in 1976. Overall, the outbreak caused >29,000 cases and >11,000 deaths and resulted in the largest known cohort of EVD survivors in history (*1*). Currently, the second-largest EVD outbreak is ongoing in the Democratic Republic of the Congo, which has had 3,340 cases and 2,207 deaths as of December 12, 2019.

During mid-2015, after considering the high number of survivors in West Africa and several episodes of EVD reemergence linked to exposure to survivors’ body fluids, the World Health Organization (WHO) adapted a strategy to manage survivors’ sequelae and mitigate the risk of resurgence (i.e., EVD cases occurring after active chains of transmission stopped) posed by viral persistence in their body fluids (*2*), such as semen, vaginal fluids, sweat, aqueous humor, urine, and breastmilk (*3*–*6*). According to WHO, an intensive integrated program was necessary to address the medical needs of survivors and the risk for virus reintroduction, ideally a program that could be integrated into existing routine health services and facilities (*2*). Therefore, the national program coordinating EVD in Guinea, in collaboration with WHO and partners, developed and implemented a survivors’ monitoring program (called SA-Ceint, derived from the French phrase “cordon sanitaire-based active surveillance”). In March 2016, when SA-Ceint was still in preparation, an episode of EVD resurgence occurred in Guinea during the WHO-endorsed 90-day period of enhanced surveillance following the declaration of the end of the outbreak. This resurgence was most likely caused by viral persistence in the semen of a survivor (*7*). Similar cases from viral persistence were previously reported in Liberia, Sierra Leone, and Guinea (*7*–*10*).

The objective of SA-Ceint was to quickly detect any new EVD cases and stop transmission early. This project was conducted by using a community-based alert system and monitoring high-risk body fluids of survivors. Here we report on the findings of this public health program.

## Methods

### Implementation of the Household-Based Alert System

During December 8, 2015–March 31, 2016, the preparatory phase of SA-Ceint occurred. We attempted to contact all EVD survivors in Guinea present in the Ministry of Health database, conducted community engagement activities, and delivered the survivors package. The communication line between survivors and the SA-Ceint team was active during April 1–September 30, 2016.

Survivors were eligible to participate in the program if they were able to show the certificate of medical clearance that they were given at release from the Ebola treatment unit (ETU). Participants received a package that included monthly allowance as well as other forms of support such as rice and flour.

The smallest structure of the monitoring program was the ring unit. This unit was built around each survivor and included his or her family or household. In each ring unit, a focal point (the survivor, or a guardian for survivors <15 years of age) and a deputy (in case the focal point was not able to perform his or her tasks) were chosen. His or her task was to report all episodes of illness in the survivor’s surroundings (i.e., episodes that involved the survivor, the immediate family, other relatives, and other persons living in proximity to the survivor). Sexually active men were prioritized as focal points because of their risk for shedding the virus in their semen. 

Each focal point was given cell phone credit to call the district health authorities regularly to relay information on episodes of illness in his or her unit. Episodes of illness, also called case alerts, were defined as deaths, cases of unexplained hemorrhage, or episodes of acute unexplained fever and vomiting, diarrhea, muscle pain, weakness or fatigue, or stomach pain. The definition of a case alert roughly corresponded to that of EVD suspected cases used in phase 3 of the Ebola response, except that phase 3 definition included 2 additional criteria: no response to treatment to common febrile diseases (e.g., malaria) and any clinical suspicion of EVD (*2*). Once the case alert was sent, this episode was investigated to collect information on treatment failure and link it to the body fluid testing results in the survivors of the concerned community, as was as any other complementary investigation considered of importance.

In each neighborhood, a platform was set up that consisted of all ring units plus the local elected representatives; this group met on a weekly basis. Its function was to resolve any type of conflict related to the program, to supervise and advise the focal point, and to inform on or validate episodes of illness occurring in the ring unit. All platform meetings were funded by the SA-Ceint program.

District data management units were responsible for communication to the central coordinating unit, the Ebola Response Coordination. Coordination of the program at this level was provided by the National Agency for Health Security, with the support of technical partners such as WHO, the US Centers for Disease Control and Prevention, the International Medical Corps, the International Federation of Red Cross and Red Crescent Societies, and the Red Cross in Guinea. A data management team was responsible for collecting and analyzing all information from health districts and laboratories. This system also monitored movement or relocation of the survivors between districts, and the ring unit was relocated according to the survivor’s displacement so that surveillance could continue. 

The National Agency for Health Security was responsible for the communication of the results of body fluid testing to the district health authorities. All male survivors >15 years of age were eligible for semen testing, and a subset of these men also was selected for urine testing. Female survivors who gave birth were eligible for body fluid testing (e.g., testing of blood, vaginal secretions, amniotic liquid, lochia, and breast milk) (Appendix Figure 1). Body fluid testing stopped after 2 negative tests by reverse-transcription PCR (RT-PCR) for the same body fluid, as recommended by WHO (*2*).

### Sampling and Laboratory Analyses

A team consisting of an epidemiologist, a nurse, and a hygienist examined and sorted the alerts and were ready to be deployed to the ring units in cases of suspected EVD to carry out an Ebola rapid diagnostic test (OraQuick Ebola rapid antigen test kit, https://www.oraquick.com) (*11*) and collect specimens for quantitative RT-PCR using appropriate personal protective equipment. The ambulance of the district was mobilized in case safe transfer of patients or bodies was needed. The team was also in charge of routinely taking biological specimens from survivors’ body fluids. Five laboratories processed biological specimens and covered the entire country of Guinea: 3 were in the capital, Conakry; 1 in the forested region of Guinea (N’zerekore); and 1 in lower Guinea (Kindia) (Appendix Figure 1). According to the standard operating procedures, which were drafted and validated before the study began, specimens were stored in an icebox at 4°C–8°C after collection and tested within 24 hours at the nearest laboratory. Breast milk specimens were tested for Ebola virus RNA by using the RealStar ZEBOV RT-PCR Kit (Altona, https://www.altona-diagnostics.com) as previously described (*12*). Seminal fluid specimens were processed as previously described (*13*), and all other body fluids were processed as previously described (*14*). Any survivor with an RT-PCR–positive semen specimen was immediately counseled and included in the JIKI trial (*15*). Eligible family, other relatives, and other people living in proximity to a survivor were enrolled in the Ebola ça Suffit! trial, aiming at evaluating efficacy and effectiveness of a vaccine against EVD (*16*).

This study was considered public health practice and was implemented following the guidelines from the WHO Ebola response phase 3 strategic document (*2*). The study was integrated in the workflow of other research projects (i.e., Postebogui, the JIKI trial, EBOSEX, and the Ebola ça Suffit! vaccination trial), all of which had been approved by the National Committee for Ethics in Research and Health before their start. All the participants signed an informed consent form at the beginning of the program.

## Results

### Outcomes of the Community-Based Alert System

We were able to retrieve information on ≈1,130/1,270 EVD survivors in Guinea, 55 of whom died after ETU discharge (late deaths) and before the program started and 140 of whom were unavailable for contact (lost to follow-up) (*17*). Excluding the 55 late deaths (and assuming no deaths occurred in the lost to follow-up category), we enrolled 1,075 of the 1,215 survivors who were known to be alive or who had been lost to follow-up (88% follow-up rate). The median starting point for follow-up was 16 months after discharge (interquartile range 14–18 months), and the median end point for follow-up was 22 months after discharge (interquartile range 20–24 months). 

In total, 9,028 immediate family members, other relatives, and other persons living in proximity to a survivor were identified (an average of 11.5 persons/ring unit), of whom 6,929 were eligible to participate in the Ebola ça Suffit! trial; 727 (10.4%) were vaccinated. Of the enrolled survivors, 47% were male and 53% female; age distribution did not vary substantially by sex (Appendix Figure 2). Seventy-nine percent of the male survivors were 15–59 years of age, whereas 78% of female survivors were 15–59 years of age. Children <15 years of age accounted for 15% of male and 16% of female survivors; persons >60 years of age accounted for 5% of the male and 6% of the female survivors. Compared with the population of Guinea, the middle age group (15–59 years of age) was overrepresented (*18*).

**Figure 1:**
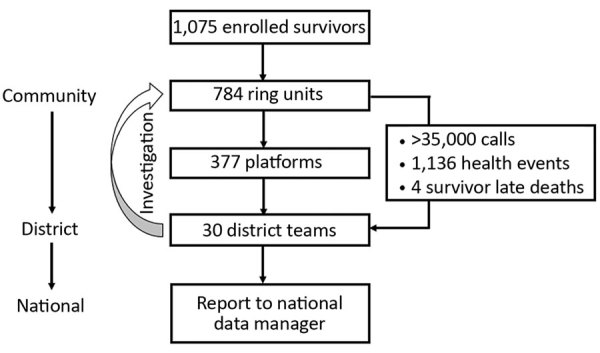
Structure of the SA-Ceint program to test body fluids from Ebola virus disease survivors to decrease risk for disease resurgence, Guinea, April–September 2016.

The structure of the program was modeled on the existing health system, from the community level to the central level. A total of 784 ring units were established at the community level, and each was represented by 1 focal point (the survivor or a legal guardian). Then, 377 platforms (open to all survivors) were created at the neighborhood level, and 30 teams were formed at the district level. During the study period, the district teams received >35,000 calls (i.e., 6 calls/month from each focal point) (Figure 1). Focal points reported 1,136 episodes of illness. Of those events, 4 were late deaths of survivors. Assuming no one experienced illness more than once, the proportion of persons who experienced an episode of illness during the 6-month period was 10% of the total. However, none of these episodes was considered to meet the definition of a suspected EVD case; therefore, no one was sent to the ETU for testing.

### Biological Monitoring of Survivors’ Body Fluids

**Figure 2:**
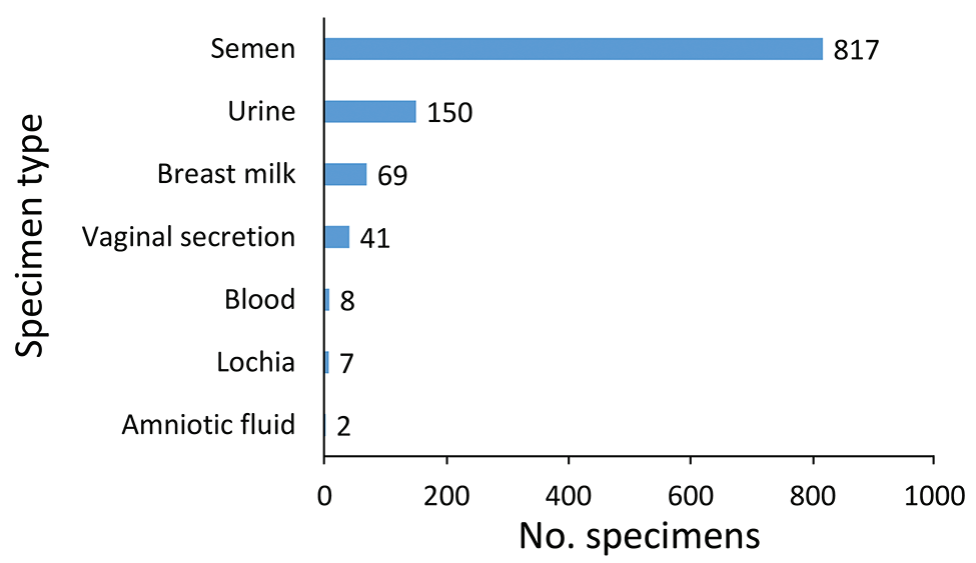
Number of specimens of body fluids from Ebola virus disease survivors tested for Ebola virus by reverse transcription PCR as part of the SA-Ceint program, Guinea, April–September 2016. All specimens tested negative except for 5 positive semen specimens from 4 survivors. Breast milk specimens were from 65 women; for all other sample types, no person had >1 samples taken.

Of the 1,094 tested specimens, most (817 [74.7%]) were semen specimens. Urine, breast milk, vaginal secretions, blood, lochia, and amniotic liquid also were tested (Figure 2); however, date of delivery for pregnant women was not recorded. The SA-Ceint program was able to test 375 (91%) of all male survivors >15 years of age, of whom 224 were tested only once (60%), 101 (27%) twice, and 50 (13%) 3 times. The lowest proportion of male survivors whose semen specimen was tested at least once was registered in the districts of Boke (33%), Kouroussa (50%), Faranah (60%), and Siguiri (78%). In all other districts, the proportion of male survivors tested was >80% (Table).

**Table:** Number of focal points and male survivors who were eligible for semen testing and were tested >1 time, by district, SA-Ceint program, Guinea, April–September 2016

District	No. focal points	No. eligible male survivors	No. male survivors tested >1 time	% Eligible survivors tested
Beyla	12	6	6	100
Boffa	6	4	4	100
Boke	6	3	1	33
Conakry	148	108	96	89
Coyah	82	36	35	98
Dubreka	35	20	20	100
Faranah	13	5	3	60
Forecariah	95	34	32	94
Fria	4	3	3	100
Gueckedou	47	18	16	89
Kankan	8	1	1	100
Kerouane	40	16	15	94
Kindia	23	10	9	90
Kissidougou	22	11	9	82
Kouroussa	5	2	1	50
Lola	11	8	8	100
Macenta	171	76	69	91
N’zerekore	24	27	26	97
Siguiri	9	9	7	78
Telimele	15	4	4	100
Yomou	4	3	3	100
Unknown	4	4	4	100
Total	784	408	372	91

Of the semen specimens tested for Ebola virus, 4 (1%) of 375 survivors were positive. In total, 5 (1%) of 817 semen specimens (2 from the same survivor) tested positive. All 4 survivors were immediately treated with favipiravir after enrollment in the JIKI trial (*15*). Three survivors’ semen specimens were positive 276, 351, and 410 days after ETU release and then negative 1 month later. The fourth survivor had his first positive semen specimen (cycle threshold value 32) 494 days after ETU release, and despite favipiravir treatment, a second positive semen specimen (cycle threshold value 23) 512 days after ETU release. His 2 following specimens tested negative.

## Discussion

We describe the experience of setting up and implementing a nationwide active surveillance program with EVD survivors in Guinea. The program aimed to mitigate the risk for EVD reintroduction from exposure to survivors’ body fluids. We were able to enroll ≈90% of the survivors in Guinea and test the semen of >90% of the enrolled male survivors >15 years of age, an unprecedented rate compared with other survivor monitoring programs (*19*–*25*).

The number of focal points was ≈75% of the enrolled survivors because some survivors were from the same household. The fact that none of the >1,000 alerts were treated as a suspected EVD episode probably means that the definition of an illness episode (which was broader than the definition of a suspected EVD case) was far from being specific. At this stage of the Ebola response, the need to investigate all alerts was more important than avoiding false-positive alerts (i.e., alerts that did not turn out to be suspected EVD cases). In fact, all 1,136 alerts were investigated. These investigations also used the available body fluid testing results. However, no alert was found to meet the definition of a suspected EVD case (i.e., none was sent to ETU for testing), in part because the case definition changed after the end of the outbreak to include cases of treatment failure, with the objective of avoiding unnecessary anxiety in the population.

We believe that the outcome of zero suspected EVD cases did reflect the low incidence of Ebola in the community at the time SA-Ceint began, when most of the survivors had recovered for >1 year. However, the outcome also probably reflected the fear of creating panic in the affected communities, because testing would otherwise not be harmful. The stigma around Ebola is surely to some extent a hurdle to the successful implementation of a program such as SA-Ceint, and our experience underscores the importance of integrating social science into outbreak response activities to enable development of risk communication strategies adapted to the local context. Still, we believe that our strategy should be considered, if the resources allow it, by other Ebola-affected countries in the future, because our approach nevertheless enabled investigation of many health alerts and would likely have detected illnesses strongly suggestive of Ebola (e.g., hemorrhagic fever).

Of the tested specimens, only 5 (from 4 survivors) were positive, and all were semen specimens. Our findings (4 positive specimens from 375 survivors tested ≥1 time) were roughly in line with the prediction from Sissoko et al. (*13*) that <1 survivor from Guinea would have an RNA-positive semen specimen by July 2016. Furthermore, RNA detection does not mean the specimen contains infectious virus. Therefore, it is not surprising to see that EVD was never suspected, despite the number of alerts, during the SA-Ceint program. At that time, the risk for reintroduction of EVD was in fact very low. However, the finding that few semen specimens were positive >1 year after survivors’ recovery confirms previous observations that male survivors’ semen must be strictly and regularly monitored after ETU release (*13*).

The SA-Ceint program was not easy to implement. We experienced delays, and by the time the program was fully implemented, evidence suggested that the risk for EVD reintroduction from survivors in Guinea into the community was already very low (*13*). This hypothesis was confirmed by our laboratory findings. Moreover, we faced reluctance among survivors to provide semen specimens because of cultural and religious reasons. The program was costly, and all survivors were financially supported; this reimbursement was possible because of the international resources that the international outbreak response mobilized.

Overall, heightened awareness in the communities and platform meetings helped us to enroll a high number of eligible survivors. Because no established network of community-based surveillance in Guinea had existed previously and because we wanted to minimize stigma among survivors, we empowered them to be the main actors in this program. We believe this was the best strategy to ensure regular reporting.

In conclusion, we successfully implemented a nationwide surveillance program for the early detection of resurgence of Ebola virus from persistently infected survivors. Our strategy could be implemented in future programs in similar contexts.

Appendix Additional information about public health program for decreasing risk for Ebola virus disease resurgence from survivors of the 2013–2016 outbreak, Guinea.

## References

[1] Coltart CEM, Lindsey B, Ghinai I, Johnson AM, Heymann DL. The Ebola outbreak, 2013-2016: old lessons for new epidemics. Philos Trans R Soc Lond B Biol Sci. 2017;372:372. 10.1098/rstb.2016.0297 28396469 PMC5394636

[2] World Health Organization. Ebola response phase 3: framework for achieving and sustaining a resilient zero [cited 2019 Dec12]. http://apps.who.int/iris/bitstream/10665/184693/1/ebola_resilientzero_eng.pdf

[3] Varkey JB, Shantha JG, Crozier I, Kraft CS, Lyon GM, Mehta AK, et al. Persistence of Ebola virus in ocular fluid during convalescence. N Engl J Med. 2015;372:2423–7. 10.1056/NEJMoa1500306 25950269 PMC4547451

[4] Rodriguez LL, De Roo A, Guimard Y, Trappier SG, Sanchez A, Bressler D, et al. Persistence and genetic stability of Ebola virus during the outbreak in Kikwit, Democratic Republic of the Congo, 1995. J Infect Dis. 1999;179(Suppl 1):S170–6. 10.1086/514291 9988181

[5] Jacobs M, Rodger A, Bell DJ, Bhagani S, Cropley I, Filipe A, et al. Late Ebola virus relapse causing meningoencephalitis: a case report. Lancet. 2016;388:498–503. 10.1016/S0140-6736(16)30386-5 27209148 PMC4967715

[6] Deen GF, Broutet N, Xu W, Knust B, Sesay FR, McDonald SLR, et al. Ebola RNA persistence in semen of Ebola virus disease survivors—final report. N Engl J Med. 2017;377:1428–37. 10.1056/NEJMoa1511410 26465681 PMC5798881

[7] Diallo B, Sissoko D, Loman NJ, Bah HA, Bah H, Worrell MC, et al. Resurgence of Ebola virus disease in Guinea linked to a survivor with virus persistence in seminal fluid for more than 500 days. Clin Infect Dis. 2016;63:1353–6. 10.1093/cid/ciw601 27585800 PMC5091350

[8] Mate SE, Kugelman JR, Nyenswah TG, Ladner JT, Wiley MR, Cordier-Lassalle T, et al. Molecular evidence of sexual transmission of Ebola virus. N Engl J Med. 2015;373:2448–54. 10.1056/NEJMoa1509773 26465384 PMC4711355

[9] Keita M, Duraffour S, Loman NJ, Rambaut A, Diallo B, Magassouba N, et al. Unusual Ebola virus chain of transmission, Conakry, Guinea, 2014–2015. Emerg Infect Dis. 2016;22:2149–52. 10.3201/eid2212.160847 27869596 PMC5189159

[10] Subissi L, Keita M, Mesfin S, Rezza G, Diallo B, Van Gucht S, et al. Ebola virus transmission caused by persistently infected survivors of the 2014–2016 outbreak in West Africa. J Infect Dis. 2018;218(suppl_5):S287–S291. 10.1093/infdis/jiy280 PMC6249578 29920602

[11] World Health Organization. WHO emergency use assessment and listing for Ebola virus disease IVDs [cited 2017 Dec 21]. http://www.who.int/diagnostics_laboratory/160324_final_public_report_ea_0023_021_00.pdf

[12] Rieger T, Kerber R, El Halas H, Pallasch E, Duraffour S, Günther S, et al. Evaluation of RealStar reverse transcription-polymerase chain reaction kits for filovirus detection in the laboratory and field. J Infect Dis. 2016;214(suppl 3):S243–9. 10.1093/infdis/jiw246 27549586 PMC5050472

[13] Sissoko D, Duraffour S, Kerber R, Kolie JS, Beavogui AH, Camara A-M, et al. Persistence and clearance of Ebola virus RNA from seminal fluid of Ebola virus disease survivors: a longitudinal analysis and modelling study. Lancet Glob Health. 2017;5:e80–8. 10.1016/S2214-109X(16)30243-1 27955791

[14] Weidmann M, Mühlberger E, Hufert FT. Rapid detection protocol for filoviruses. J Clin Virol. 2004;30:94–9. 10.1016/j.jcv.2003.09.004 15072761

[15] Sissoko D, Laouenan C, Folkesson E, M’Lebing A-B, Beavogui A-H, Baize S, et al.; JIKI Study Group. Experimental treatment with favipiravir for Ebola virus disease (the JIKI trial): a historically controlled, single-arm proof-of-concept trial in Guinea. PLoS Med. 2016;13:e1001967. 10.1371/journal.pmed.1001967 26930627 PMC4773183

[16] Henao-Restrepo AM, Camacho A, Longini IM, Watson CH, Edmunds WJ, Egger M, et al. Efficacy and effectiveness of an rVSV-vectored vaccine in preventing Ebola virus disease: final results from the Guinea ring vaccination, open-label, cluster-randomised trial (Ebola Ça Suffit!). Lancet. 2017;389:505–18. 10.1016/S0140-6736(16)32621-6 28017403 PMC5364328

[17] Keita M, Diallo B, Mesfin S, Marega A, Koumpingnin NY, N’Faly M, et al. Subsequent mortality of Ebola virus disease survivors in Guinea: a nationwide retrospective cohort study. Lancet Infect Dis. 2019;19:1202–8. 10.1016/S1473-3099(19)30313-5 31494017

[18] Ministry of Health of Guinea. National Health Development Plan 2015–2024 [cited 2019 Dec 12]. http://www.nationalplanningcycles.org/sites/default/files/country_docs/Guinea/plan_national_developpement_sanitaire_2015-2024_guinee_fin.pdf

[19] Soka MJ, Choi MJ, Baller A, White S, Rogers E, Purpura LJ, et al. Prevention of sexual transmission of Ebola in Liberia through a national semen testing and counselling programme for survivors: an analysis of Ebola virus RNA results and behavioural data. Lancet Glob Health. 2016;4:e736–43. 10.1016/S2214-109X(16)30175-9 27596037

[20] Purpura LJ, Soka M, Baller A, White S, Rogers E, Choi MJ, et al. Implementation of a national semen testing and counseling program for male Ebola survivors—Liberia, 2015–2016. MMWR Morb Mortal Wkly Rep. 2016;65:963–6. 10.15585/mmwr.mm6536a5 27632552

[21] Etard J-F, Sow MS, Leroy S, Touré A, Taverne B, Keita AK, et al.; Postebogui Study Group. Multidisciplinary assessment of post-Ebola sequelae in Guinea (Postebogui): an observational cohort study. Lancet Infect Dis. 2017;17:545–52. 10.1016/S1473-3099(16)30516-3 28094208

[22] Subtil F, Delaunay C, Keita AK, Sow MS, Touré A, Leroy S, et al.; Postebogui Study Group. Dynamics of Ebola RNA persistence in semen: a report from the Postebogui cohort in Guinea. Clin Infect Dis. 2017;64:1788–90. 10.1093/cid/cix210 28329169

[23] Deen GF, McDonald SLR, Marrinan JE, Sesay FR, Ervin E, Thorson AE, et al.; Sierra Leone Ebola Virus Persistence Study Group. Implementation of a study to examine the persistence of Ebola virus in the body fluids of Ebola virus disease survivors in Sierra Leone: Methodology and lessons learned. PLoS Negl Trop Dis. 2017;11:e0005723. 10.1371/journal.pntd.0005723 28892501 PMC5593174

[24] Abad N, Malik T, Ariyarajah A, Ongpin P, Hogben M, McDonald SLR, et al.; Sierra Leone Ebola Virus Persistence Study Group. Development of risk reduction behavioral counseling for Ebola virus disease survivors enrolled in the Sierra Leone Ebola Virus Persistence Study, 2015-2016. PLoS Negl Trop Dis. 2017;11:e0005827. 10.1371/journal.pntd.0005827 28892490 PMC5593175

[25] Sneller MC, Reilly C, Badio M, Bishop RJ, Eghrari AO, Moses SJ, et al.; PREVAIL III Study Group. A longitudinal study of Ebola sequelae in Liberia. N Engl J Med. 2019;380:924–34. 10.1056/NEJMoa1805435 30855742 PMC6478393

