# Supplementary material for: Public Health Program for Decreasing Risk for Ebola Virus Disease Resurgence from Survivors of the 2013–2016 Outbreak, Guinea
Source: Emerg Infect Dis. 2020 Feb;26(2):206–11. doi: 10.3201/eid2602.191235 (PMC6986820; doi:10.3201/eid2602.191235)
Supplement: Appendix — Additional information about public health program for decreasing risk for Ebola virus disease resurgence from survivors of the 2013–2016 outbreak, Guinea. [file 19-1235-Techapp-s1.pdf]

Article DOI: https://doi.org/10.3201/eid2602.191235

Public Health Program for Decreasing Risk for Ebola Virus Disease Resurgence from Survivors of the 2013–2016 Outbreak, Guinea

Appendix

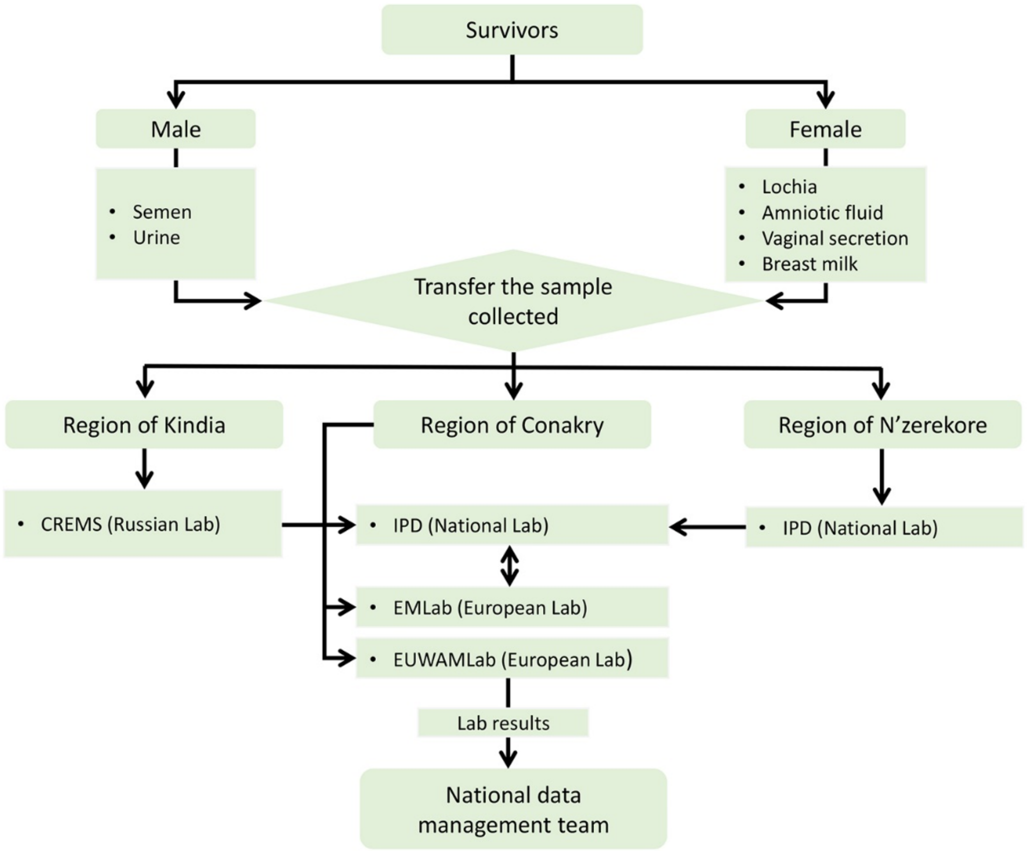

Appendix Figure 1. Workflow of the body fluids' specimens of survivors tested during the SA-Ceint program.

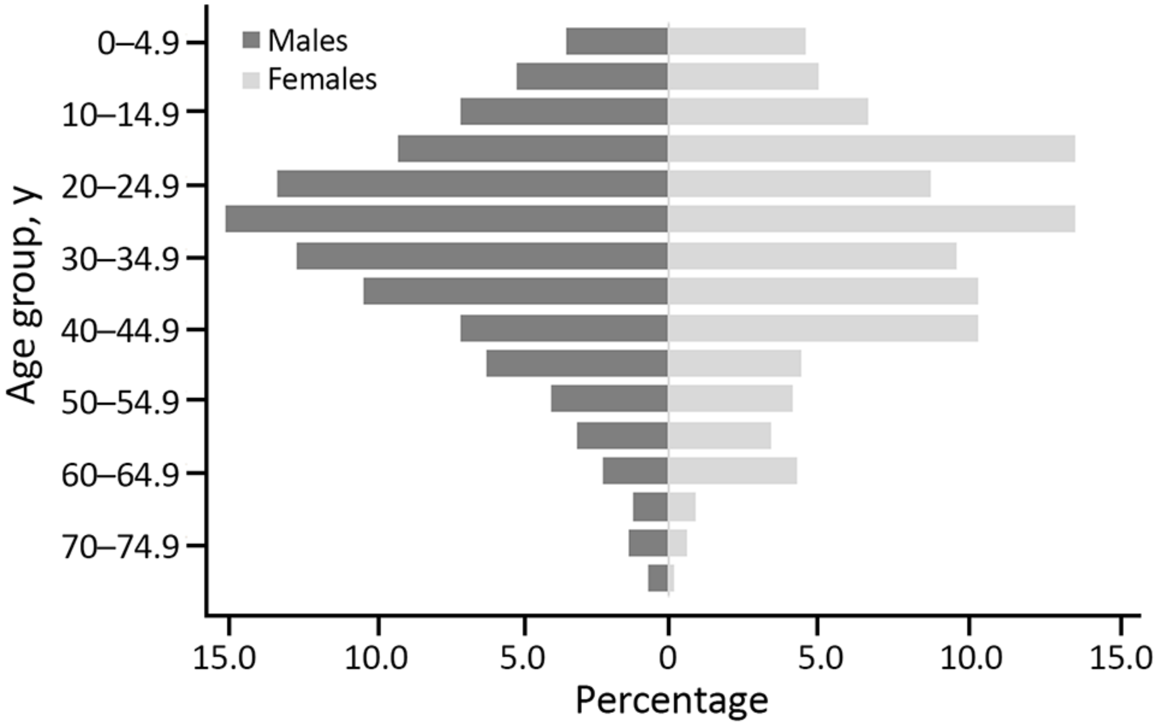

Appendix Figure 2. Age and sex distribution of the EVD survivors enrolled in the SA-Ceint program (n = 1,075).
